# Replacing the phthalimide core in thalidomide with benzotriazole

**DOI:** 10.1080/14756366.2021.2024525

**Published:** 2022-02-28

**Authors:** Mikhail Krasavin, Andrey Bubyrev, Alexander Kazantsev, Christopher Heim, Samuel Maiwald, Daniil Zhukovsky, Dmitry Dar’in, Marcus D. Hartmann, Alexander Bunev

**Affiliations:** a Institute of Chemistry, Saint Petersburg State University, Saint Petersburg, Russia; b Department of Protein Evolution, Max Planck Institute for Developmental Biology, Tübingen, Germany; c Medicinal Chemistry Center, Togliatti State University, Togliatti, Russia

**Keywords:** Cereblon, immunomodulatory drugs, phthalimide, benzotriazole, diazo compounds, carbene N-H insertion

## Abstract

The advent of proteolysis-targeting chimaeras (PROTACs) mandates that new ligands for the recruitment of E3 ligases are discovered. The traditional immunomodulatory drugs (IMiDs) such as thalidomide and its analogues (all based on the phthalimide glutarimide core) bind to Cereblon, the substrate receptor of the CRL4A^CRBN^ E3 ligase. We designed a thalidomide analogue in which the phthalimide moiety was replaced with benzotriazole, using an innovative synthesis strategy. Compared to thalidomide, the resulting “benzotriazolo thalidomide” has a similar binding mode, but improved properties, as revealed in crystallographic analyses, affinity assays and cell culture.

## Graphical Abstract

**Figure:**
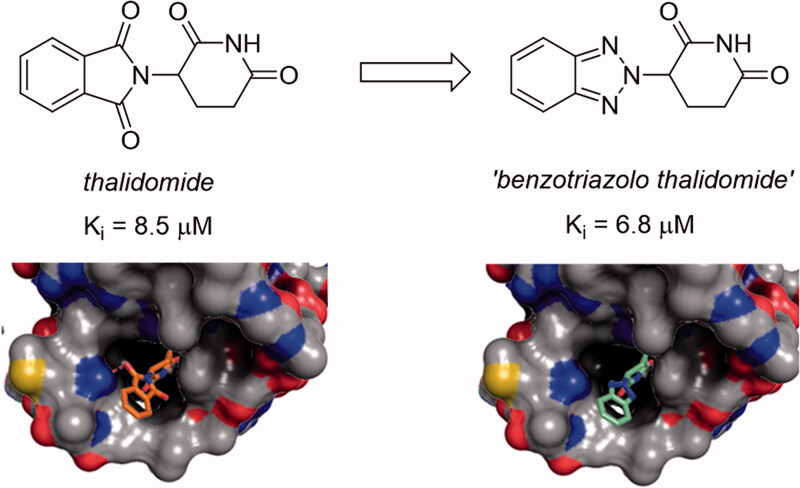

## Introduction

The approach to eliminating dysregulated proteins *via* targeted protein degradation is rapidly gaining momentum as an alternative to small-molecule inhibitors^1^. One of the most promising and powerful molecular tools to achieve that are the so-called proteolysis-targeting chimaeras (PROTACs) in which two recruiter moieties are joined with a linker^2^. One is a ligand of the protein of interest (POI, i.e. the one to be degraded) and the other a ligand of an E3 ligase. Once the two proteins (POI and E3 ligase) are brought in proximity by the PROTAC molecule, this triggers poly-ubiquitination of the POI by the E3 ligase, which makes the former a client for proteasomal degradation^3^. Cereblon (CRBN) is one of the most important E3 ligases that has been employed for PROTAC development^4^ so far. The ligand space of CRBN mostly includes phthalimide-based thalidomide (**1**) and its analogs^5^. In addition to these, structural requirements for CRBN ligand binding have recently been elucidated^6^.

The binding mode of thalidomide (**1**) includes a hydrogen bond between one of the phthalimide carbonyl groups and a conserved asparagine residue of the protein, which contributes to the affinity between the two molecules^7^. However, the other phthalimide carbonyl is not involved in any specific interactions, not even with water molecules, and thus represents an unsatisfied polar group that potentially lowers the binding energy. We hypothesised that replacing the phthalimide core with benzotriazole, thereby removing both carbonyl groups, would possibly eliminate the possibility of hydrogen bonding with the conserved asparagine, but also rid the molecule of the “dissatisfied” carbonyl group. As the net result, the affinity to CRBN may be retained, assuming that the introduced triazole nitrogen is less unfavoured than the carbonyl group due to its decreased polarity with only a single free electron pair. Herein, we report on the verification of this hypothesis.

## Results and discussion

**Scheme 1.**
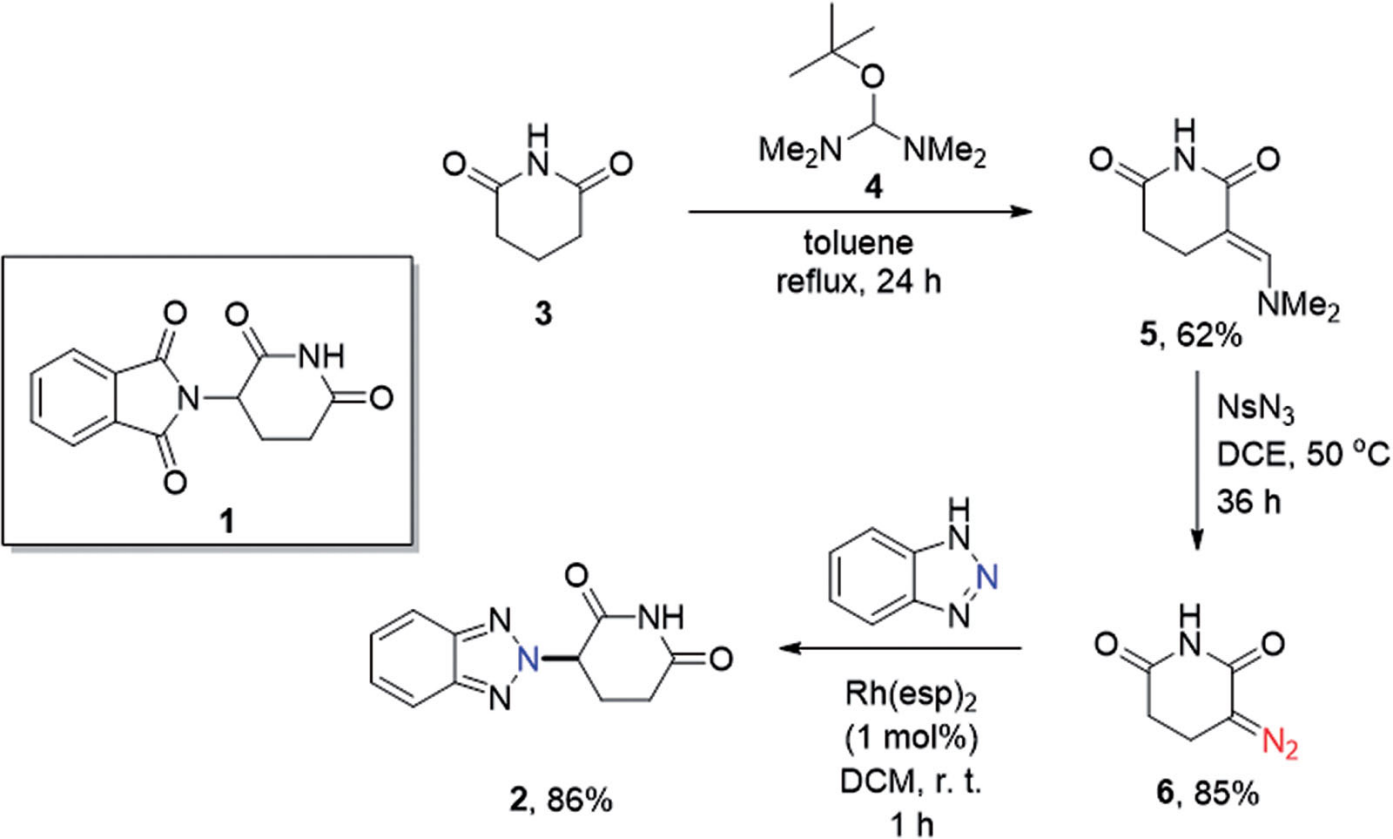
Synthesis of ‘benzotriazolo thalidomide’ **2**.

The synthesis of the benzotriazole analogue **2** of thalidomide was achieved as detailed below. Commercially available glutarimide **3** was (dimethylamino)methylenated at the α-position using the Brederick’s reagent (**4**)^8^. The resulting derivative **5** readily entered the Regitz diazo transfer reaction^9^ with 4-nitrophenylsulfonyl azide (NsN_3_) to give hitherto undescribed 3-diazopiperidine-2,6-dione (**6**) in excellent yield. α-Diazocarbonyl compounds were recently established to regioselectively alkylate benzotriazoles at *N^2^* when activated as Rh(II) carbenes^10^. Indeed, when α-diazoglutarimide (**6**) was activated by Rh(II) espionate (bis[rhodium(α,α,α′,α′-tetramethyl-1,3-benzenedipropionic acid)]) (1 mol%) and reacted with benzotriazole, desired ‘benzotriazolo thalidomide’ **2** was obtained in excellent yield and complete regioselectivity (Scheme 1).

To our delight, when evaluated for affinity to the thalidomide-binding domain of human CRBN in comparison to thalidomide (**1**, K_i_ 8.5 ± 0.8 µM), using the recently reported thermophoresis-based assay^11^, the benzotriazolo analogue **2** displayed an improvement in affinity with a K_i_ value of 6.8 ± 1.6 µM.

**Figure 1.**
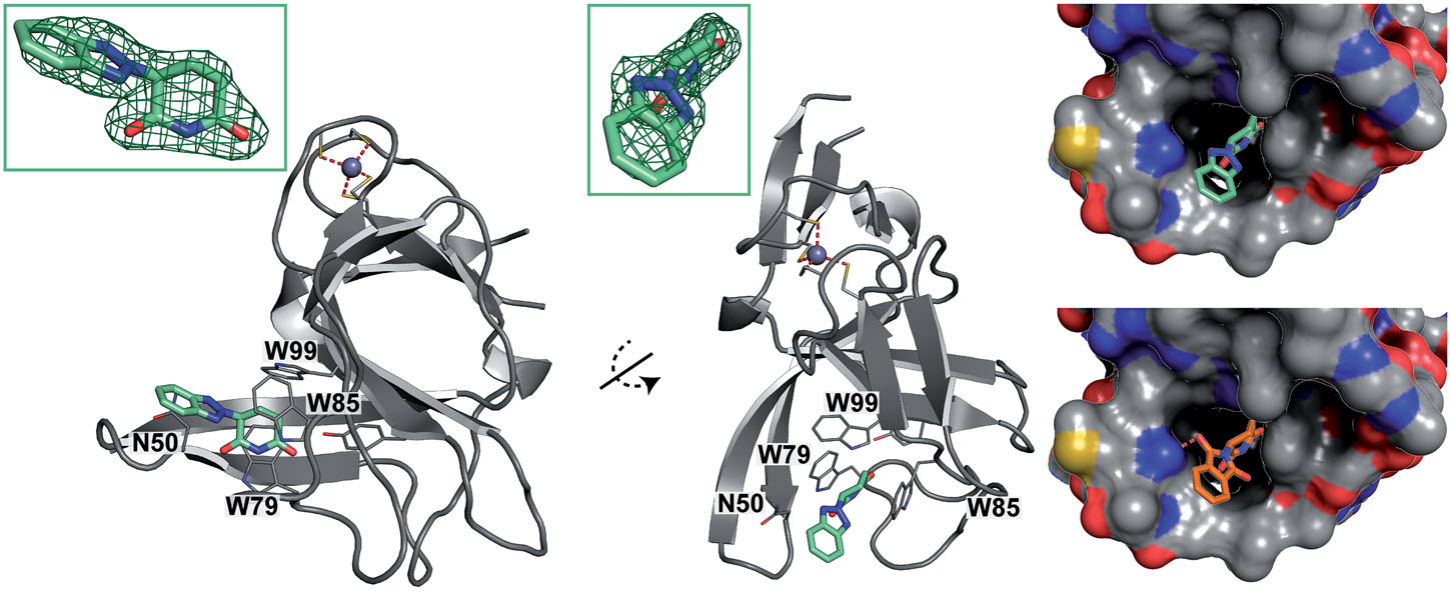
Binding mode of ‘benzotriazolo thalidomide’ (**2**) compared to thalidomide. Left: two views of **2** bound to MsCI4 with an F_O_-F_C_ omit map contoured at 4σ. Three tryptophan residues and a conserved asparagine residue of the binding site are indicated. Of note, the asparagine does not form interactions with **2**. Right: The binding of **2** compared to thalidomide in surface representation coloured by atom type. The hydrogen bond of thalidomide to the conserved asparagine is indicated. Residue numbering according to the MsCI4 sequence.

To gain insight into the binding mode of **2**, we employed our previously established crystal soaking system based on the bacterial CRBN homologue *Magnetospirillum gryphiswaldense* Cereblon Isoform 4 (MsCI4)^6^. The obtained crystal structure revealed that **2** binds in the same overall orientation as thalidomide, but lacks any specific hydrogen-bonding interactions with the target other than those mediated by the glutarimide moiety (Figure 1)^12^. None of the benzotriazole nitrogen is involved in hydrogen bonds, not even to water, suggesting that the retained affinity could indeed be due to their lower polarity as compared to the ‘unsatisfied’ carbonyl groups in thalidomide.

**Figure 2.**
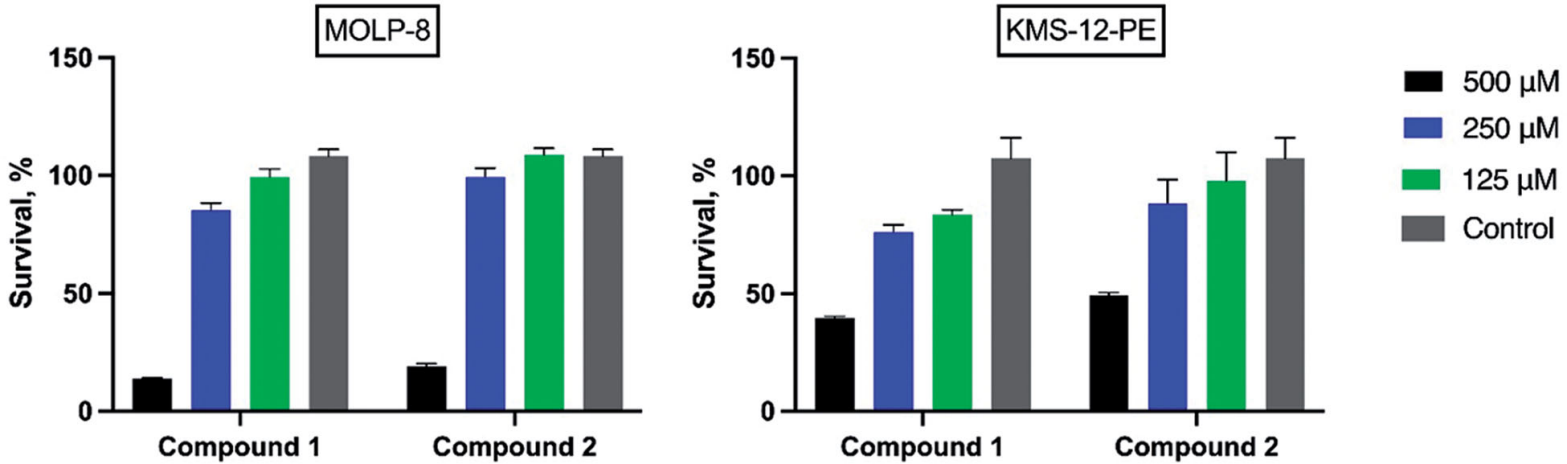
Cytotoxicity profile of compounds **1** and **2** against two multiple myeloma cell lines.

Usage of **2** in PROTAC design mandates that a functionalised version of it (akin to lenalidomide^13^ or pomalidomide^14^) is developed. Before taking steps into that direction, we were keen to determine the cytotoxicity and apoptosis-inducing profile of “benzotriazolo thalidomide” **2**, in comparison to thalidomide (**1**) itself. Figure 2 shows the cytotoxicity of the two compounds towards MOLP-8^15^ and KMS-12-PE^16^ multiple myeloma cell lines, which clearly demonstrates the absence of any appreciable cytotoxicity at concentrations as high as 250 µM.

As to the apoptosis-inducing ability (evaluated by flow cytometry in MOLP-8 cells), compound **2** showed a clear advantage compared to thalidomide (**1**): at 300 µM, it preserved a substantially higher population of live cells (Table 1). This clearly shows the promise of the ‘benzotriazolo thalidomide’ scaffold reported herein for the future use in the design of PROTACs.

**Table 1.** Apoptosis induction by compounds **1** and **2** (300 µM, MOLP-8 cells, 48 h incubation time).

Compound	Live cells, %	Early apoptosis, %	Late apoptosis, %	Dead, %
Control	90.07 ± 2.15	3.26 ± 1.56	5.86 ± 1.63	0.82 ± 1.02
**1**	57.36 ± 2.1	20.47 ± 1.03	21.14 ± 0.68	0.97 ± 0.45
**2**	70.57 ± 1.54	21.94 ± 1.64	6.78 ± 0.53	0.7 ± 0.39

## Conclusion

In summary, we have described a promising novel benzotriazolo analogue of thalidomide. Despite the absence of the carbonyl group involved in hydrogen bonding with CRBN, it retained affinity, likely due to the relief from the other, ‘dissatisfied’ carbonyl group. These assumptions are corroborated by the crystal structure of the complex between **2** and CRBN. Compound 2 is distinctly non-cytotoxic towards multiple myeloma cell lines (at concentrations as high as 250 µM) and preserves more live cell population in apoptosis-induction experiments, compared to thalidomide (**1**). Development of a functionalised version of **2** for the use in the PROTAC design is highly desirable and is currently underway in our laboratories.

## Supplementary Material

Supplemental Material
