# Supplementary material for: Replacing the phthalimide core in thalidomide with benzotriazole
Source: J Enzyme Inhib Med Chem. 2022 Feb 28;37(1):527–30. doi: 10.1080/14756366.2021.2024525 (PMC8890552; doi:10.1080/14756366.2021.2024525)

Supplementary data for

Replacing the Phthalimide Core in Thalidomide with Benzotriazole

Mikhail Krasavin,* Andrey Bubyrev, Alexander Kazantsev, Christopher Heim, Samuel Maiwald, Daniil Zhukovsky, Dmitry Dar'in, Marcus D. Hartmann, Alexander Bunev

Contents

Experimental procedures and characterization data	S2-S3
Copies of ^{1}H and $^{13}C\{^{1}H\}$ NMR spectra	S4-S6
Microscale Thermophoresis	S7
Protein Crystallography	S7-S8
Description of cellular assays	S9-S10

Experimental procedures and characterization data

General considerations.

All reagents were obtained from commercial sources and used without further purification. Tetrahydrofuran and dichloromethane were distilled over suitable drying agents. Mass spectra were recorded with a Bruker Maxis HRMS-ESI-qTOF spectrometer (electrospray ionization mode). NMR data were recorded with Bruker Avance 400 spectrometer (400.13 MHz for ^{1}H, 100.61 MHz for ^{13}C) in $CDCl_3$ and were referenced to residual solvent proton peaks (δ_H = 7.28) and solvent carbon peaks (δ_C = 77.0).

(*E*)-3-((Dimethylamino)methylene)piperidine-2,6-dione (5)

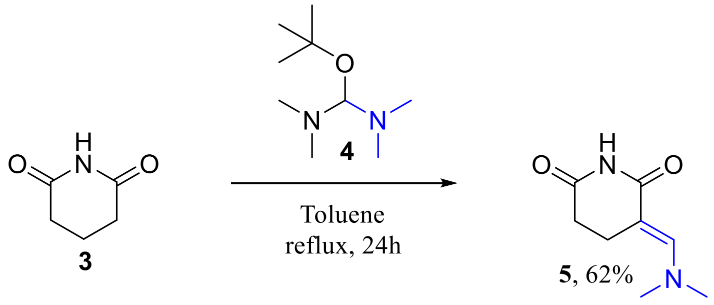

A solution of glutarimide (543 mg, 4.8 mmol) and Brederek's reagent (1.09 ml, 5.3 mmol, 1.1 equivalent) in toluene (15 ml) was refluxed for 24 h under Ar and evaporated. The reaction mixture was dissolved in chloroform and washed with water (1 x 25 ml) and brine (1 x 25 ml). The organic layer was dried over Na_2SO_4. The target compound was purified by column chromatography using a mixture of chloroform - methanol (2%) as eluent.

Yield 530 mg, 62%. Yellow solid. ^{1}H NMR (500 MHz, $CDCl_3$) δ 8.48 (s, 1H), 3.05 (s, 6H), 2.78 (t, J = 7.1 Hz, 2H), 2.46 (t, J = 7.1 Hz, 2H). ^{13}C NMR (126 MHz, $CDCl_3$) δ 173.22, 169.21, 150.23, 91.10, 43.39, 31.94, 19.97. HRMS (ESI/Q-TOF) m/z: $[M+H]^+$ Calcd for $C_8H_{12}N_2O_2$ 169.0972; Found 169.0971.

3-Diazopiperidine-2,6-dione (6)

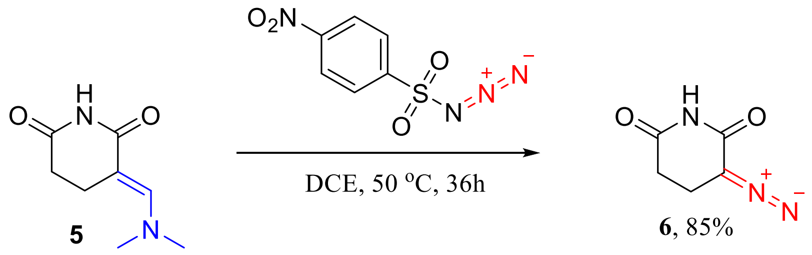

To a solution of enamine (500 mg, 3.0 mmol) in 1,2-dichloroethane (10 ml) was added 4-nitrophenylsulfonyl azide (814 mg, 3.6 mmol, 1.2 equivalent) and the resulting solution was stirred

for 36 hours at 50 °C under Ar and evaporated. The target compound was purified by column chromatography using a mixture of chloroform - methanol (3%) as eluent.

Yield 355 mg, 85%. Light green solid. ^{1}H NMR (500 MHz, DMSO-d_6) δ 10.6 (s, 1H), 2.9 (t, J = 7.2 Hz, 2H), 2.6 (t, J = 7.2 Hz, 2H). ^{13}C NMR (126 MHz, DMSO-d_6) δ 172.22, 167.06, 54.71, 30.80, 16.18. HRMS (ESI/Q-TOF) m/z: $[M+H]^+$ Calcd for $C_5H_5N_3O_2$ 140.0455; Found 140.0457.

3-(2*H*-Benzo[*d*][1,2,3]triazol-2-yl)piperidine-2,6-dione (2)

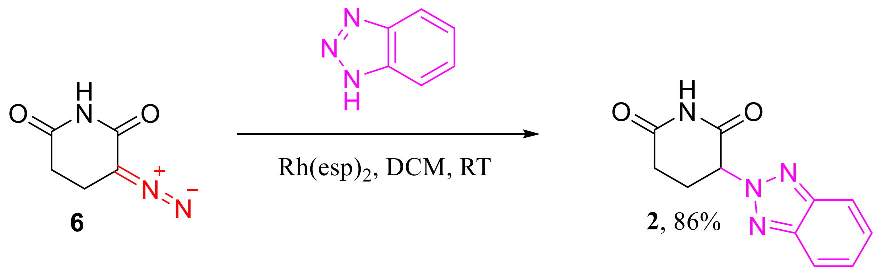

To a solution of diazo glutarimide (0.75 mmol) and benzotriazole (0.75 mmol) in dichloromethane (10 ml) was added bis[rhodium(α,α,α′,α′-tetramethyl-1,3-benzenedipropionic acid)] (6 mg, 1 mol%) and the resulting solution was stirred for three hour at room temperature. The reaction mixture was centrifuged and dried under atmosphere.

Yield 149 mg, 86%. White solid. ^{1}H NMR (400 MHz, DMSO-d_6) δ 11.28 (s, 1H), 7.97 (dd, J = 6.6, 3.1 Hz, 2H), 7.49 (dd, J = 6.6, 3.1 Hz, 2H), 6.28 – 6.05 (m, 1H), 3.01 – 2.68 (m, 4H). HRMS (ESI/Q-TOF) m/z: $[M+H]^+$ Calcd for $C_{11}H_{10}N_4O_2$ 231.0877; Found 231.0878. ^{13}C NMR (126 MHz, DMSO-d_6) δ 172.90, 169.44, 144.15, 127.34, 118.50, 65.30, 30.85, 24.90.

Copies of ^{1}H and $^{13}C\{^{1}H\}$ NMR spectra.

^{1}H and ^{13}C NMR spectra of compound **5**

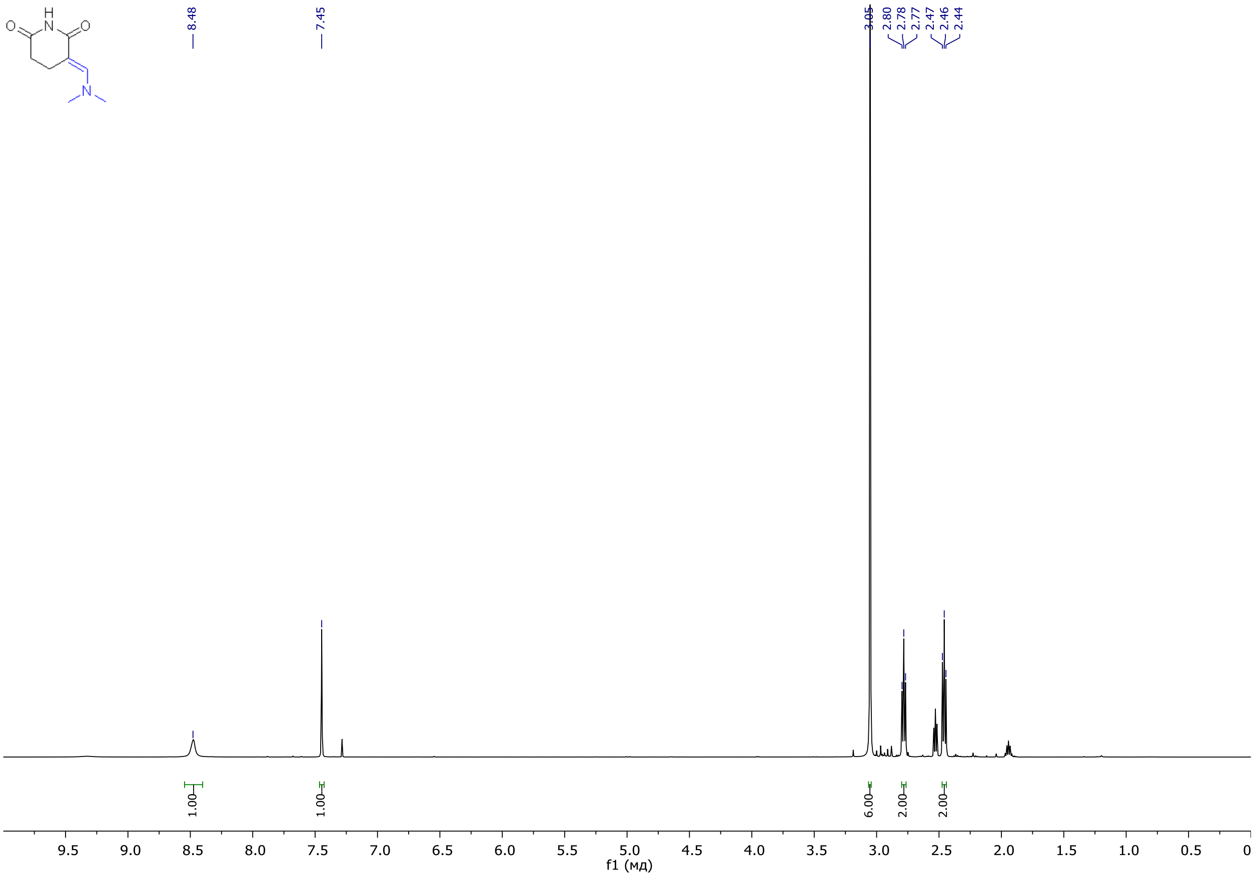

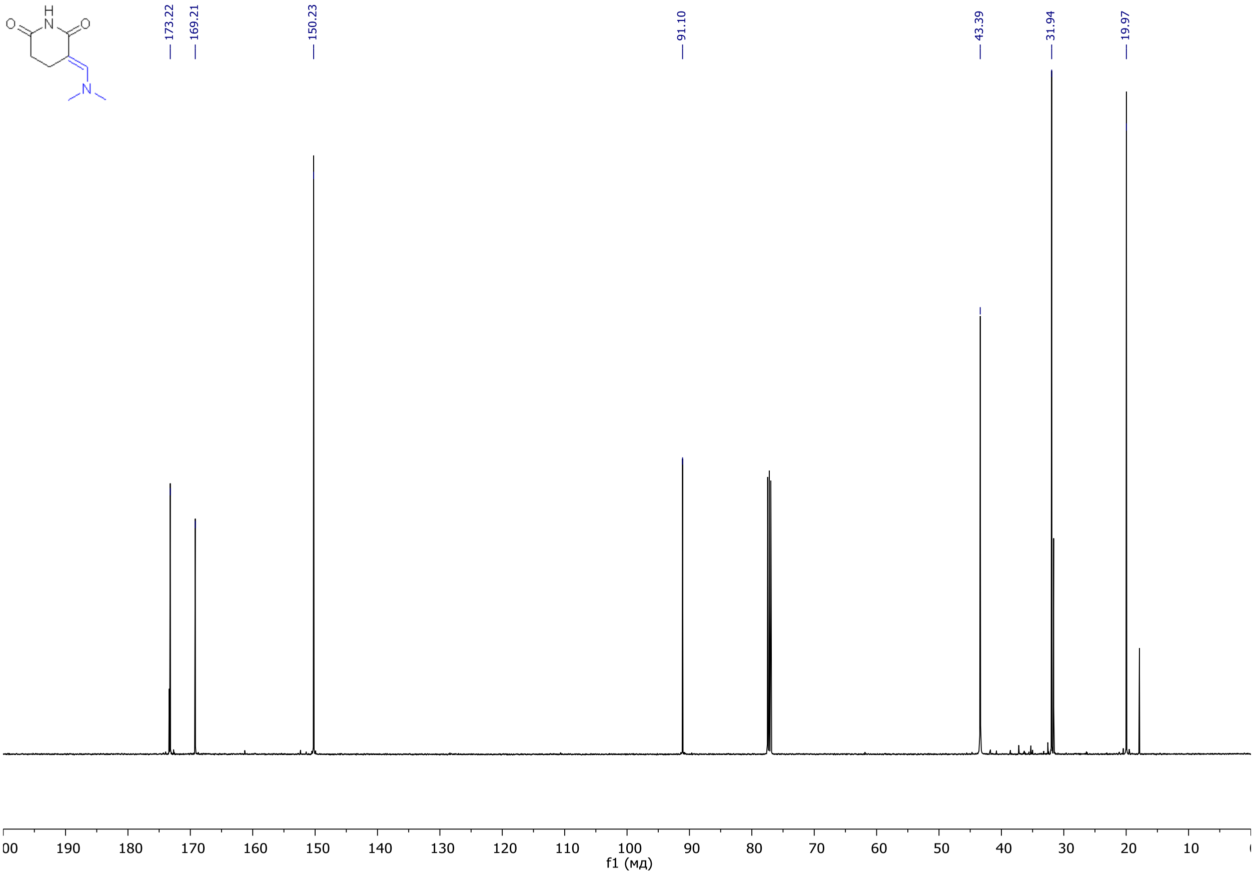

^{1}H and ^{13}C{^{1}H} NMR spectra of compound **6**

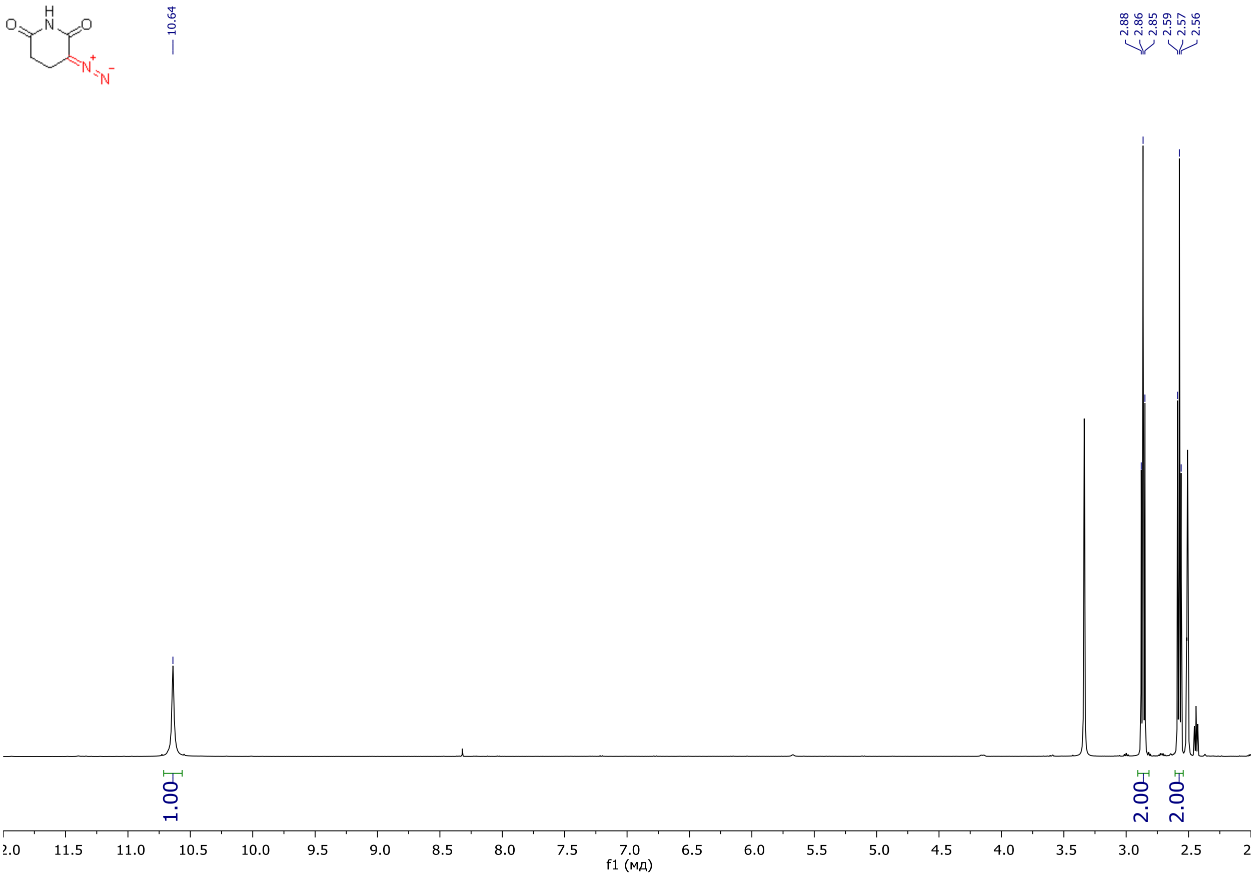

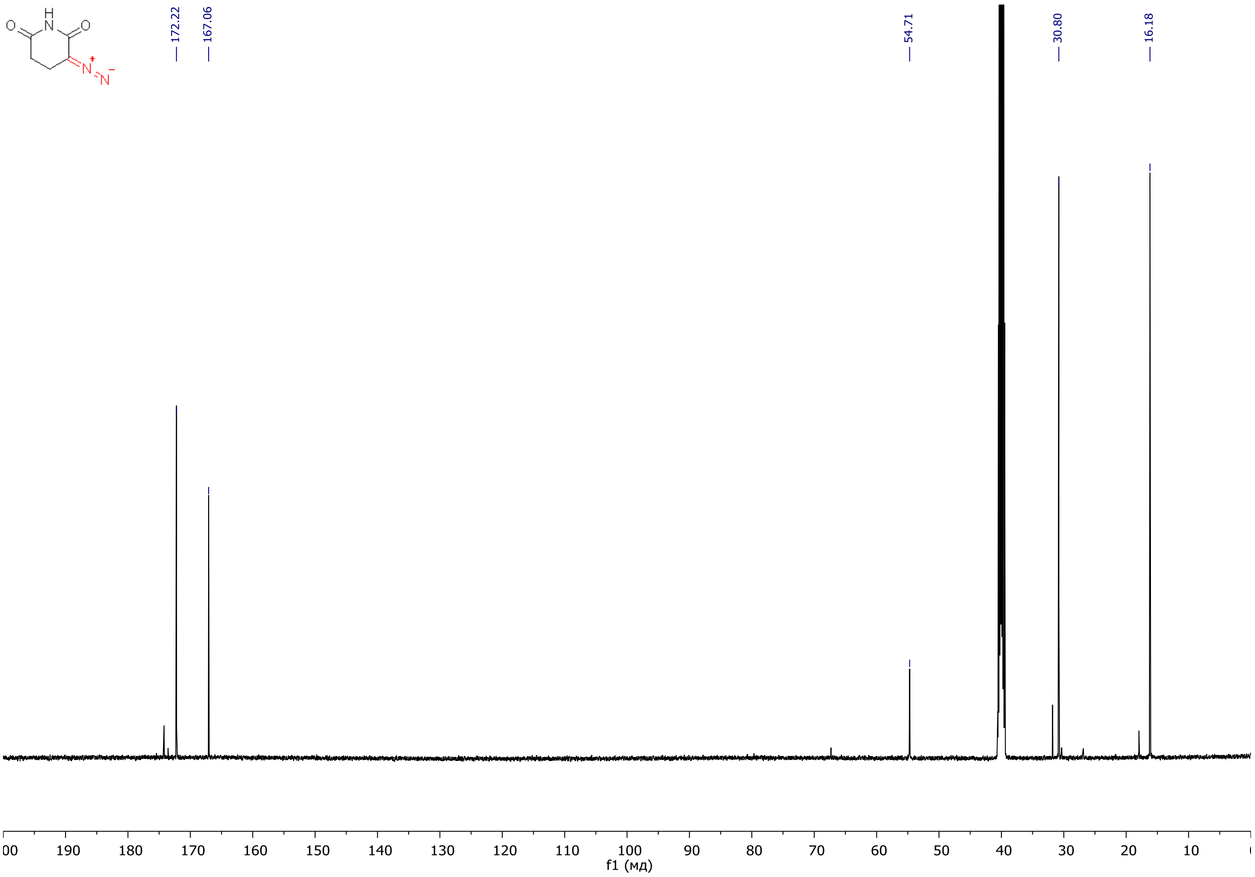

^{1}H and ^{13}C{^{1}H} NMR spectra of compound **2**

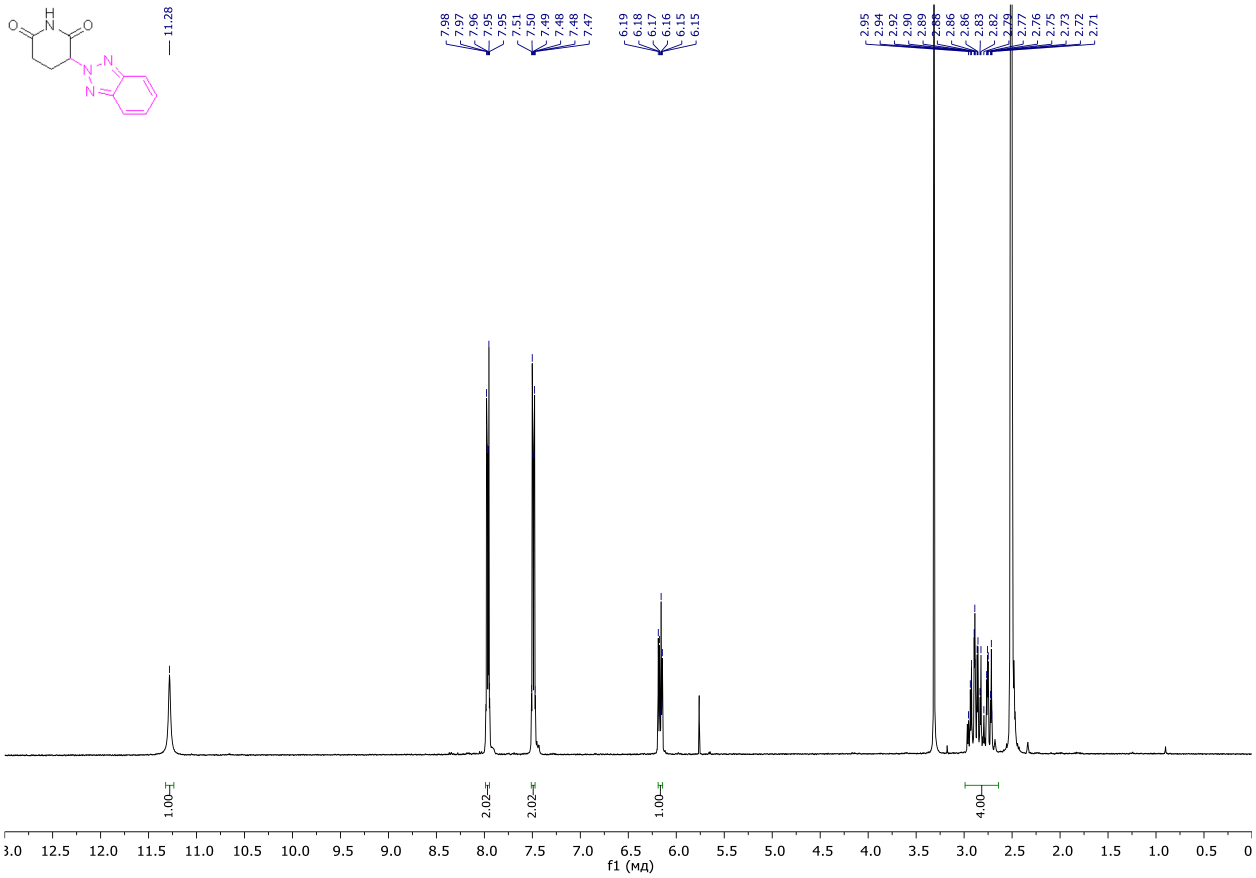

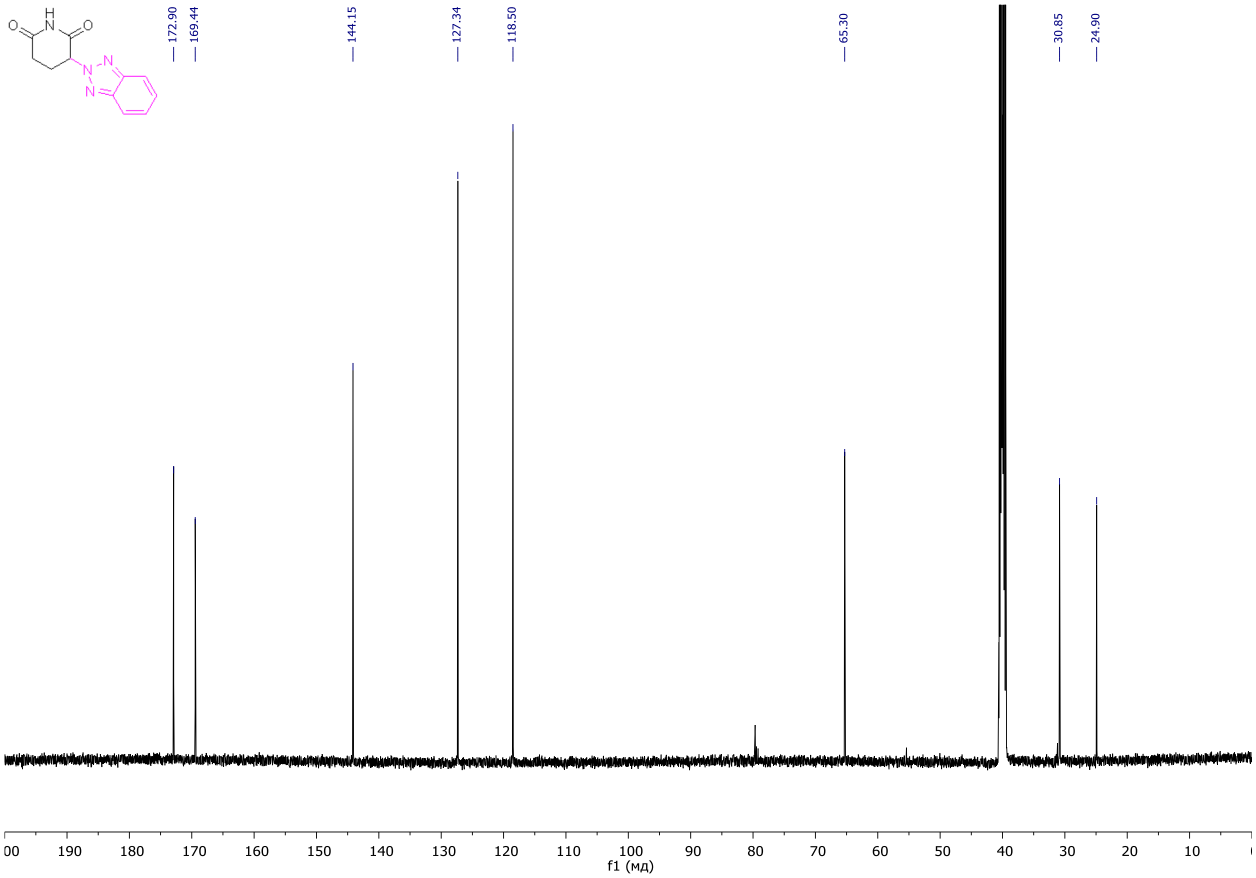

Microscale Thermophoresis

Binding affinities of thalidomide (**1**) and its benzotriazole analog (**2**) to the thalidomide-binding domain of human CRBN (hTBD) were determined as described previously.[1] In short, a 16-point 1:1 dilution series of the compound in DMSO was diluted 1:100 in ddH_2O and then mixed with protein:reporter stock to final concentrations of 10 µM hTBD and 200 nM BODIPY-uracil. Measurements were performed on a Monolith NT.115 with a Nano BLUE detector (NanoTemper Technologies), using 20% excitation power, MST power set to medium and temperature control at 25 °C. Data at on-time 20 s were analyzed using Prism 9 (GraphPad).

X-ray Crystallography

Crystals of MsCI4 were grown via the vapor diffusion technique at 20 °C in sitting-drops by mixing 200 nL MsCI4 protein solution (17 mg/mL) supplemented with 3 mM **1** and 200 nL 0.4 M $(NH4)_2HPO4$ solution.[2] Big crystals belonging to the orthorhombic space group $P2_12_12_1$ appeared within a few days and were used for soaking experiments. To this end, crystals were transferred into fresh ground solution drops spiked with **2** and incubated for 72h. Crystals were cryoprotected with reservoir solution supplemented with 70% sodium malonate and flash-cooled in liquid nitrogen. Diffraction data was collected at 100 K on beamline X10SA at the Swiss Light Source using an EIGER2 16 M detector. Data was processed and scaled to 1.99 Å using XDS[3] and the structure solved using difference Fourier methods: Refinement with REFMAC5[4] and cyclic modelling using *Coot*[5] using PDB 4V2Y as a starting model clearly located **2** bound to two of the three MsCI4 molecules in the asymmetric unit. Molecular figures were generated using PyMOL (The PyMOL Molecular Graphics System, Version 2.5.0 Schrödinger, LLC.). Data collection and refinement statistics are summarized in Table S1. The structure was deposited in the Protein Data Bank (PDB) under the accession code 7PJK.

---

[1] S. Maiwald, C. Heim, B. Hernandez Alvarez, M. D. Hartmann, Sweet and Blind Spots in E3 Ligase Ligand Space Revealed by a Thermophoresis-Based Assay, ACS Med. Chem. Lett. 2020, 12, 74-81.
[2] C. Heim, D. Pliatsika, F. Mousavizadeh, K. Bär, B. Hernandez Alvarez, A. Giannis, M. D. Hartmann, J. Med. Chem. 2019, 62, 6615-6629.
[3] W. Kabsch, Acta Crystallogr. D Biol. Crystallogr. 2010, 66, 125-132.
[4] G. N. Murshudov, A. A. Vagin, E. J. Dodson, Acta Crystallogr. D Biol. Crystallogr. 1997, 53, 240-255.
[5] P. Emsley, K. Kowtan, Acta Crystallogr. D Biol. Crystallogr. 2004, 60, 2126-2132.

Table S1: Data collection and refinement. Values in parenthesis refer to the highest resolution shell.

**Data collection**	
Space group	P $2_12_12_1$
**Unit Cell**	
a, *b*, *c* (Å)	56.78, 59.44, 88.19
α, β, γ (°)	90.00, 90.00, 90
Resolution range (Å)	35.41-1.99 (2.11-1.99)
Redundancy	12.8 (13.0)
Completeness (%)	99.7 (98.3)
R merge (%)	9.5 (181)
CC(1/2)	99.9 (58.3)
$I/\sigma(I)$	14.9 (1.25)
**Refinement**	
No. reflections (total/test)	21050 (2046)
No. atoms	2168
Protein	2071
Ligand	37
Solvent	60
R_{work}	0.21
R_{free}	0.25

Cell culture

Multiple myeloma cell line MOLP-2 and KMS-12-PE were purchased from the DSMZ. Cells were maintained in RPMI-1640 (Gibco, UK) supplemented with 20% fetal bovine serum (FBS, Gibco, UK), penicillin (100 UI mL^{-1}), streptomycin (100 µg mL^{-1}) and GlutaMax (2 mM, Gibco, UK). All cells line cultivation under a humidified atmosphere of 95% air/5% CO_2 at 37 °C. The number of viable cells was determined by trypan blue exclusion.

MTT assay

All examined cells were diluted with the growth medium to 3.0×10^5 cells per mL and the aliquots (15×10^3 cells per 50 µL) were placed in individual wells in white 96-multiplates (Nunc, USA). Triplicate wells were treated with test compounds starting at 500.0 µM concentration and diluted at various concentrations or DMSO (Sigma, USA) as control with final concentration 0.1%. Plates were incubated for 72 h at 37 °C in 5% CO_2 atmosphere. After incubation, the cells were then treated with 100 µL CellTiter-Glo® One Solution (Promega, USA). The plates were shaken for 10 min. The luminescence was determined using a microplate reader GloMax Multi+ (Promega, USA). Each of the tested compounds was evaluated for cytotoxicity in three separate experiments.

Apoptosis assay

For the detection of apoptosis, the cells were plated at 6-well culture plates (Eppendorf, Germany). The exposed cells were placed at 37 °C in a 5% CO_2 incubator for 48 h. The cultured cells were washed twice with PBS and resuspended in 1× binding buffer (AnnexinV-FITC kit, Invitrogen, USA) at a concentration 1×10^6 mL^{-1}. Annexin-FITC (5 µL) and propidium iodide (PI, 2 µL) were added to 100 µL of the cell suspension and incubated for 15 min at room temperature (25 °C) in the dark. After incubation 400 µL of 1× binding buffer was added to each tube and the stained cells were analyzed within 1 h using CytoFlex (Beckham Culture, USA) and CytExpert 2.1 program. Since, Annexin V FITC staining precedes the loss of membrane integrity that accompanies the later stage identified by PI, Annexin FITC positive, PI negative indicates early apoptosis, while the viable cells are Annexin V FITC negative, PI negative. The cells that are in late apoptosis, or dead are both Annexin V FITC and PI positive. Each of the tested compounds was evaluated for induction apoptosis in three separate experiments.

MOLP-8 cells stained by Annexin-FITC and propidium iodide analyzed by flow cytometry

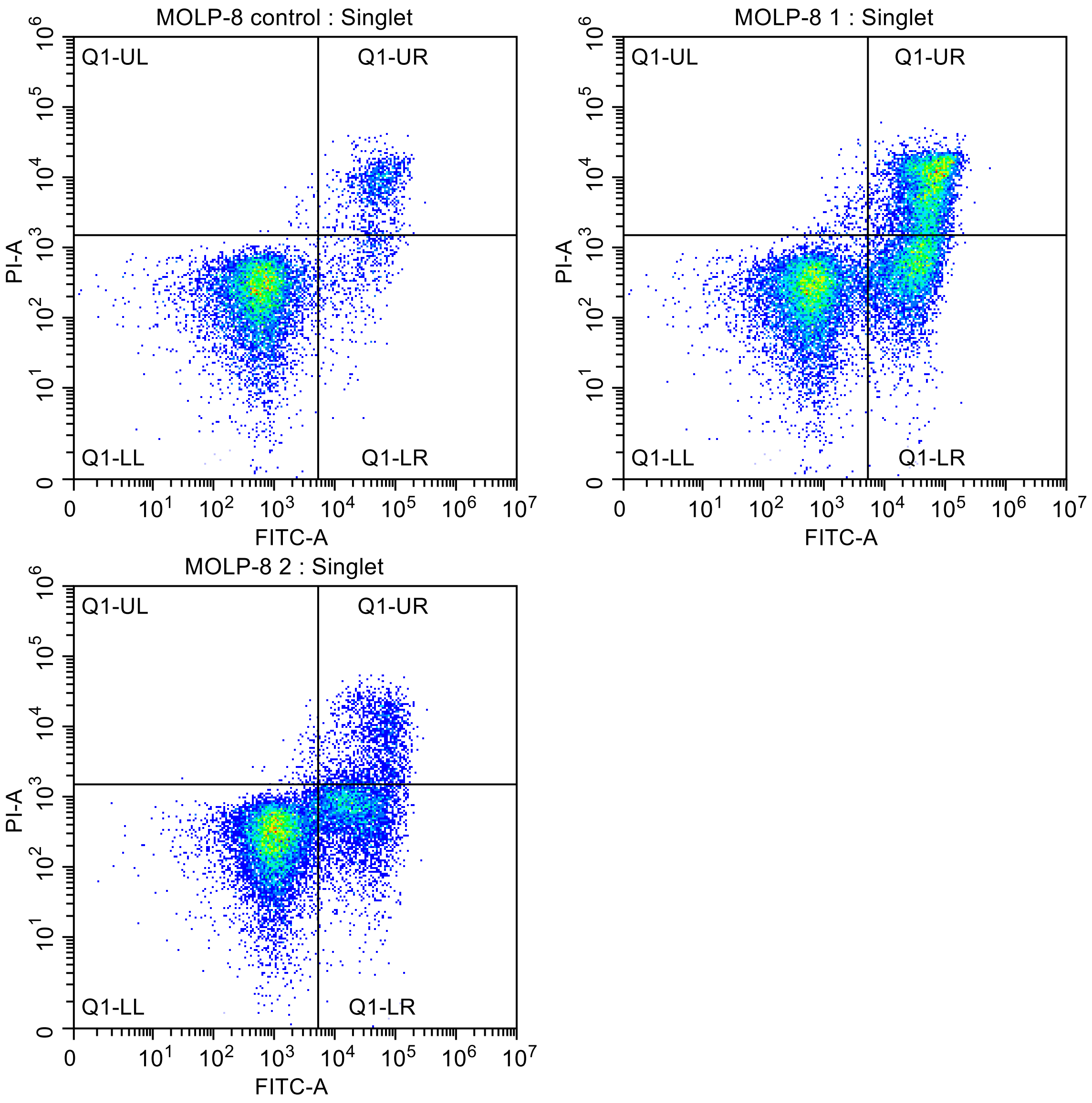

Supplement: Supplemental Material [file IENZ_A_2024525_SM4075.pdf]
